# Atopic dermatitis, short stature, skeletal malformations, hyperimmunoglobulin E syndrome, hypereosinophilia and recurrent infections: a case report

**DOI:** 10.1186/1752-1947-7-253

**Published:** 2013-11-07

**Authors:** Salvatore Leonardi, Martina Filippelli, Valentina Costanzo, Novella Rotolo, Mario La Rosa

**Affiliations:** 1 Department of Medical and Pediatric Science, University of Catania, Via S. Sofia 78, 95100, Catania, Italy

**Keywords:** Atopic dermatitis, Hypereosinophilia, Hyperimmunoglobulin E, Short stature, Skeletal malformations

## Abstract

**Introduction:**

We report an interesting clinical case which could represent a new syndrome never described previously in the literature.

**Case presentation:**

A 15-year-old Caucasian boy presented to our institution with recurrent respiratory infections, severe atopic dermatitis, short stature and skeletal malformations. Laboratory tests showed a high level of immunoglobulin E, hypereosinophilia with a normal white blood cell count and a low level of somatomedin C. The patient had had atopic dermatitis resistant to treatment since the age of 6 months. His height did not increase despite receiving cyclic therapy with recombinant growth hormone.

**Conclusion:**

We hypothesized the presence of several diseases not confirmed by any genetic tests. Our patient could have an unknown disease. Further research is needed to identify this possible new syndrome.

## Introduction

We report a case of a child with recurrent respiratory and skin infections, severe atopic dermatitis, short stature and skeletal abnormalities of unknown origin.

## Case presentation

A 15-year-old Caucasian boy was admitted to our department for short stature (less than the third percentile), low weight (less than the third percentile), severe atopic dermatitis and skeletal abnormalities. The family history was unremarkable. He was born at the 34th week of pregnancy and was adequate for gestational age with normal psychomotor development. Since the age of 6 months, he has had treatment-resistant (antihistamines and steroids) dermatitis with recurrent skin infections. His skin prick test was highly positive for respiratory and food allergens (*Olea*, *Parietaria*, cat and dog dander, dust mites, egg and soy). He also had experienced bronchial asthma with persistent cough and recurrent respiratory tract infections since the age of 4 years.

**Figure 1:**
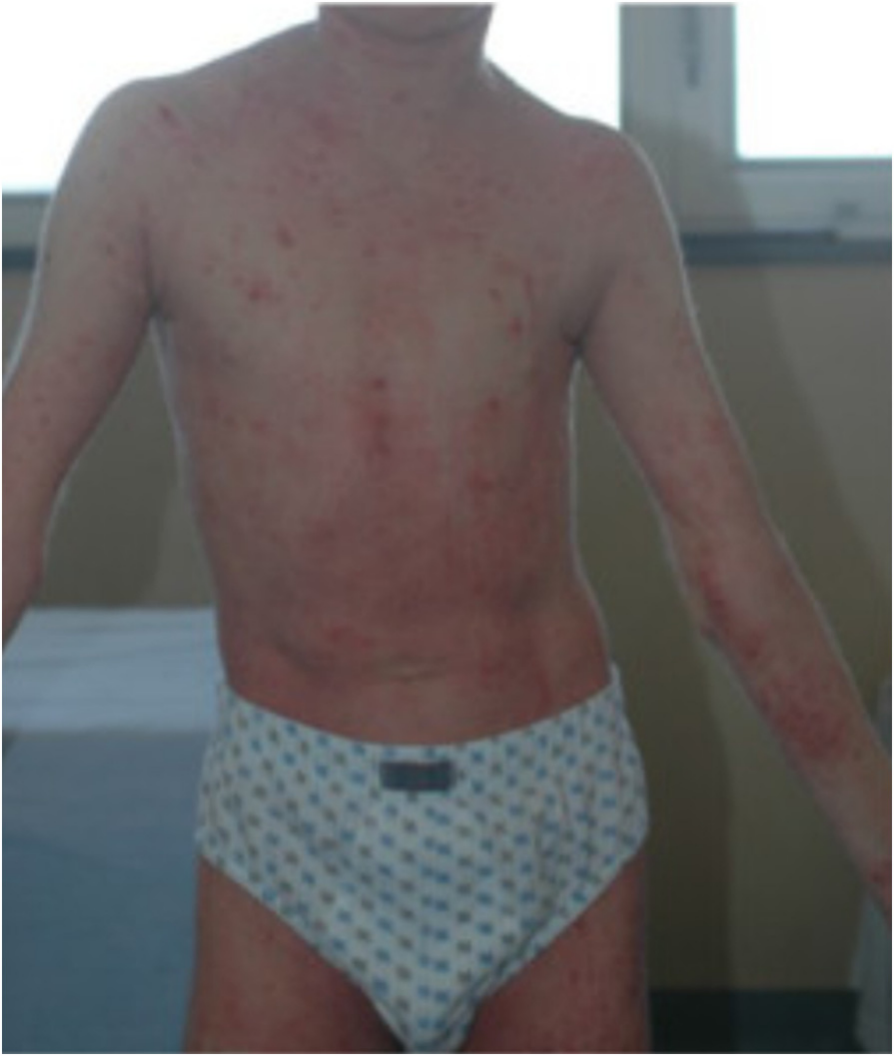
Atopic dermatitis.

The patient came to our attention with growth retardation, respiratory symptoms (rhinorrhea and cough), severe eczema on the whole skin with scratching lesions (Figure 1) and skeletal abnormalities, including excavated chest, asymmetry of the shoulder girdle, cervical kyphosis, scoliosis, lower-limb dysmetria, pelvic asymmetry and left clubfoot disease.

Immunological evaluation showed hypereosinophilia (2160μl) with a normal white blood cell count (9.680/mm^3^) and an abnormal increase in serum immunoglobulin E (IgE) level (37.308IU/ml). Other serum immunoglobulin levels were IgA (834mg/dl), IgG (1310mg/dl) and IgM (266mg/dl). Lymphocyte subsets showed CD19+ B cells 20%, CD3+ T cells 71%, CD3+ CD8+ T cells 30% and CD3+ CD4+ T cells 37%.

To identify the possible causes of his growth failure, we performed other laboratory tests, including those for hepatic and renal function, thyroid hormones, human growth hormone (hGH), somatomedin C and celiac disease. As the basal value of somatomedin C was extremely low (109ng/ml; normal range, 247ng/ml to 482ng/ml), we performed an arginine test to evaluate the hGH release from the pituitary gland after stimulation with arginine (Table 1). The laboratory test showed a hGH level less than 10ng/ml after 60 minutes. His sex hormones, prolactin and cortisol levels were all normal.

**Table 1:** Human growth hormone levels after arginine stimulation test

Growth hormone at 0 minutes	3.60ng/ml
Growth hormone at 15 minutes	0.31ng/ml
Growth hormone at 30 minutes	7.50ng/ml
Growth hormone at 60 minutes	2.30ng/ml

We also performed an X-ray of his left hand and wrist (called “bone age X-ray”) to evaluate the maturity of his bones. The results showed a bone age (9 years) younger than his chronological age. We performed a brain magnetic resonance imaging scan to look for any changes or disturbances in the area of the hypothalamus and the pituitary gland, but the findings were normal.

**Figure 2:**
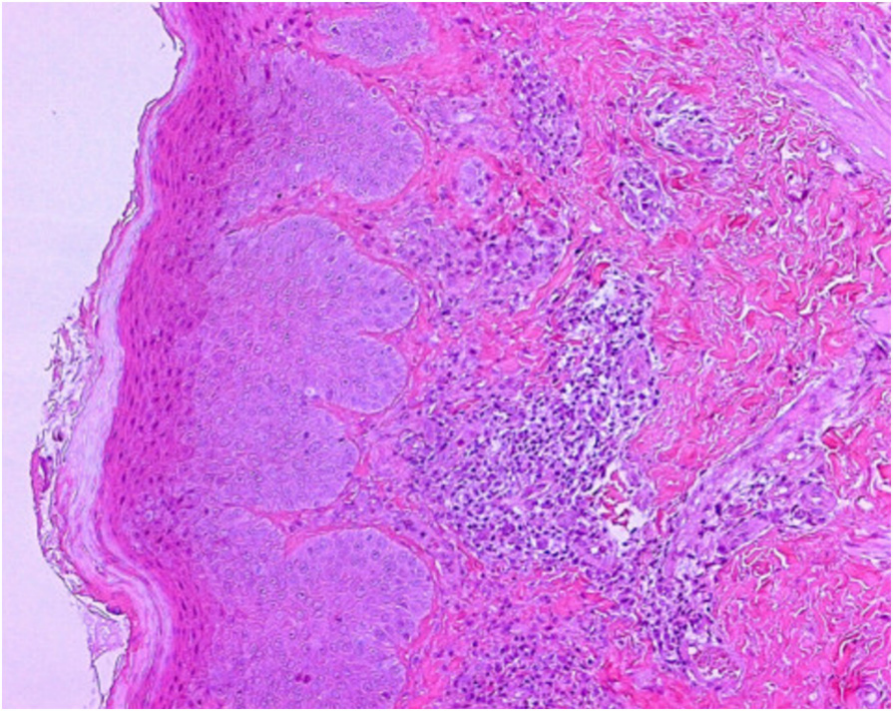
Histological image of the epidermal layer with a mild lymphohistiocytic and granulocytic infiltrate, predominantly eosinophilic.

**Figure 3:**
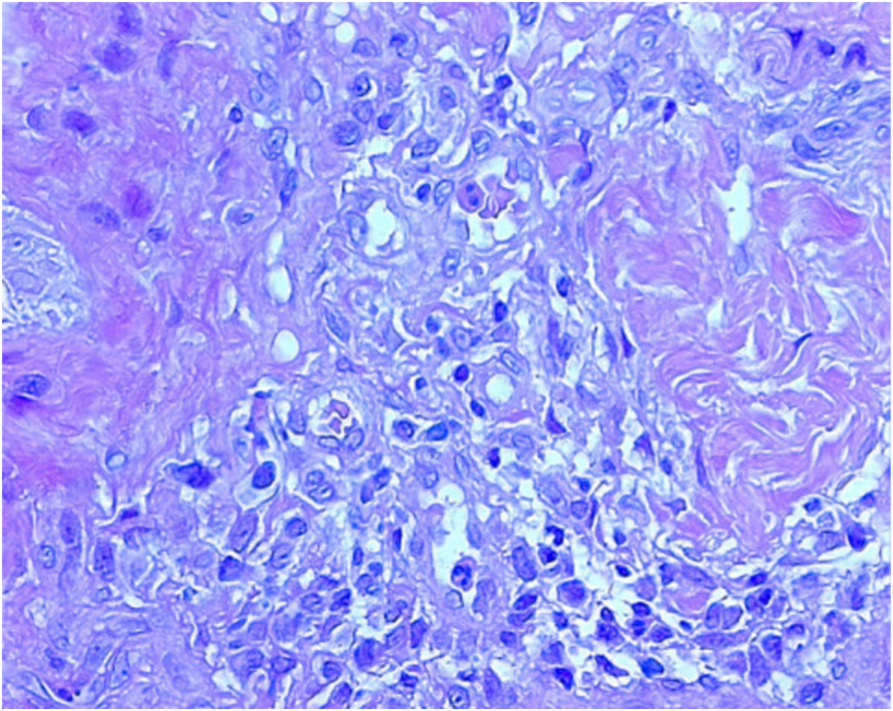
**Enlargement of Figure**2**showing lymphohistiocytic and granulocytic infiltrate.**

The spine and pelvis X-rays showed accentuation of upper dorsal kyphosis with asymmetry of clavicles and accentuation of lordosis at the lumbosacral transition. The pelvis was rotated with asymmetry of the bicrestoiliaca line. The femoral right head was raised 7mm compared to the left side. The chest X-ray showed a diffuse reinforcement of bronchovascular pattern as a consequence of both chronic inflammatory infiltrate and recurrent respiratory infections. The main skin biopsy histopathological findings were hyperkeratosis, acanthosis and spongiosis with a chronic inflammatory infiltrate composed mainly of lymphocytes, mast cells and eosinophils (Figures 2 and 3).

On the basis of the patient findings and laboratory tests, including molecular genetic tests, we postulated different diagnoses, but no one could explain our patient’s presentation. We investigated Noonan syndrome [1], but we did not find mutations of the genes involved (*PTPN11*, *KRAS*, *SOS1*, *NRAS*, *RAF1*,*CBL*, *SHOC2*, *BRAF*, *MAP2K1*, *HRAS*, *NF1* and *SPRED1*); celiac disease [2]; polyendocrine diseases for short stature [3]; food allergies [4-6], hypereosinophilic syndromes [7], but lymphocyte phenotyping on peripheral blood was normal; immunodysregulation, polyendocrinopathy, enteropathy, X-linked (IPEX) [8], but genomic sequence analysis of the *FOXP3* gene was negative for its mutations; autosomal-dominant hyperimmunoglobulin E syndrome (also known as Job syndrome), but genetic testing did not show any mutation of the *STAT3* gene [9]; and hyperimmunoglobulin E syndrome associated with Tyk2 deficiency, but the molecular analysis was normal [10].

The aim of the treatment was to reduce the symptoms of atopic dermatitis, including prevention of the patient’s recurrent skin infections and improvement in his growth. The treatment with recombinant growth hormone was suspended because of the lack of an adequate response to therapy, with a height increase of only 4cm over more than one year. The skin manifestations of eczema were treated with histamine 1 antagonists, topical and oral steroids, tacrolimus and cyclosporine. Unfortunately, the patient did not have any improvement. At the one-year follow-up examination, the patient presented with recurrent skin and respiratory infections, despite the use of prophylactic antibiotics. No other improvement in the patient’s clinical condition was observed.

## Conclusion

We report detailed clinical history and laboratory features of an interesting case of a 15-year-old boy with an unknown disease. We suspected different diagnoses such as Noonan syndrome, celiac disease, polyendocrine diseases for short stature, food allergies, hypereosinophilic syndromes, IPEX, autosomal-dominant hyperimmunoglobulin E syndrome and hyperimmunoglobulin E associated with Tyk2 deficiency. Unfortunately, none of the laboratory tests performed, including genetic analysis, confirmed our suspicions. Our report could be a case of a new syndrome. Further studies and genetic research are needed to support this hypothesis.

## Consent

Written informed consent was obtained from the patient’s legal guardian for publication of this case report and accompanying images. A copy of the written consent is available for review by the Editor-in-Chief of this journal.

## Competing interests

The authors declared that they have no competing interests.

## Authors’ contributions

SL did the primary writing of the case report, with substantial contributions from MF and VC. All authors contributed to revisions of the manuscript and approved the final version for publication.
